# The Etiology of Winter Hay Fever in Texas

**Published:** 1917-06

**Authors:** Sam N. Key

**Affiliations:** Austin, Texas


### THE ETIOLOGY OF WINTER HAY FEVER IN TEXAS.

BY DR. SAM N. KEY, AUSTIN, TEXAS.

The usual conception of hay fever is that it occurs only in the spring and fall of the year, the former being caused by the pollen of the grasses and the fall type chiefly by the pollen of the ragweed. However, there occurs in various parts of Texas a winter hay fever. This variety has never been previously described nor its cause ascertained. It begins about Christmas, or a little before, and lasts until about the middle of February. Coming as it does in mid-winter, the disease is frequently mistaken for a persistent "cold."

In conjunction with Dr. W. Scheppegrell, President of the American Hay Fever Prevention Association, the author by a series of observations and experiments has determined the cause of this winter hay fever to be the pollen of the male mountain cedar. The male cedar is readily recognized during the winter hay-fever season by the brownish tint the cones containing the pollen give the foliage of the tree.

Inasmuch as so little can be offered in the way of treatment, the knowledge of the cause of this type of hay fever is of practical value. The fact that the disease can be entirely avoided by change of abode to a place where mountain cedar is not found is of value to some. While the cedar is small and may traverse considerable distance, by avoiding the proximity of the cedars and by destroying such male cedar as may be on the premises of susceptible persons, the severity of the attacks will be lessened. Then, too, the knowledge of the cause of winter hay fever opens the road to pollen therapy, which at present is our chief hope for the prevention and cure of hay fever.

---

A leprosy case has been reported at Dallas. The patient has been placed under strict quarantine.

